# Malignant Arrhythmogenic Role Associated with *RBM20*: A Comprehensive Interpretation Focused on a Personalized Approach

**DOI:** 10.3390/jpm11020130

**Published:** 2021-02-15

**Authors:** Paloma Jordà, Rocío Toro, Carles Diez, Joel Salazar-Mendiguchía, Anna Fernandez-Falgueras, Alexandra Perez-Serra, Monica Coll, Marta Puigmulé, Elena Arbelo, Ana García-Álvarez, Georgia Sarquella-Brugada, Sergi Cesar, Coloma Tiron, Anna Iglesias, Josep Brugada, Ramon Brugada, Oscar Campuzano

**Affiliations:** 1 Cardiology Department, Hospital Clinic, University of Barcelona-IDIBAPS, 08036 Barcelona, Spain; pjordab@clinic.cat (P.J.); elenaarbelo@secardiologia.es (E.A.); ANAGARCI@clinic.cat (A.G.-A.); josep@brugada.org (J.B.); 2 Medicine Department, School of Medicine, University of Cadiz, 11001 Cadiz, Spain; rociotorogreen@gmail.com; 3 Biomedical Research and Innovation Institute of Cadiz (INiBICA), 11001 Cadiz, Spain; 4 Cardiovascular Diseases Research Group Bellvitge Biomedical Research Institute (IDIBELL) Hospitalet de Llobregat, 08001 Barcelona, Spain; carles.diezlopez@gmail.com (C.D.); jsalazarmg@gmail.com (J.S.-M.); 5 Advanced Heart Failure and Heart Transplant Unit Department of Cardiology Bellvitge University Hospital Hospitalet de Llobregat, 08001 Barcelona, Spain; 6 Cardiovascular Genetics Center, University of Girona-IDIBGI, 17001 Girona, Spain; afernandez@gencardio.com (A.F.-F.); aperez@idibgi.org (A.P.-S.); mcoll@gencardio.com (M.C.); mpuigmule@gencardio.com (M.P.); annai@brugada.org (A.I.); 7 Centro de Investigación Biomédica en Red Enfermedades Cardiovasculares (CIBERCV), 28029 Madrid, Spain; 8 Pediatric Arrhythmias, Inherited Cardiac Diseases and Sudden Death Unit, Cardiology Department, Hospital Sant Joan de Déu, University of Barcelona, 08950 Barcelona, Spain; georgia@brugada.org (G.S.-B.); sergi.cesar@gmail.com (S.C.); 9 Medical Science Department, School of Medicine, University of Girona, 17001 Girona, Spain; 10 Cardiology Service, Hospital Josep Trueta, University of Girona, 17001 Girona, Spain; colomatiron@gmail.com

**Keywords:** sudden cardiac death, arrhythmia, dilated cardiomyopathy, genetics, *RBM20*

## Abstract

The *RBM20* gene encodes the muscle-specific splicing factor RNA-binding motif 20, a regulator of heart-specific alternative splicing. Nearly 40 potentially deleterious variants in *RBM20* have been reported in the last ten years, being found to be associated with highly arrhythmogenic events in familial dilated cardiomyopathy. Frequently, malignant arrhythmias can be a primary manifestation of disease. The early recognition of arrhythmic genotypes is crucial in avoiding lethal episodes, as it may have an impact on the adoption of personalized preventive measures. Our study performs a comprehensive update of data concerning rare variants in *RBM20* that are associated with malignant arrhythmogenic phenotypes with a focus on personalized medicine.

## 1. Introduction

Sudden cardiac death (SCD) may be the first manifestation of an inherited arrhythmogenic disease. In consequence, the early identification of individuals at risk and the adoption of personalized measures may help to prevent a lethal episode. In this review, we focus on *RBM20*, a gene that is associated with highly aggressive arrhythmogenic phenotypes in patients diagnosed with dilated cardiomyopathy (DCM) in spite of an apparently normal heart or scattered heart alterations.

## 2. Dilated Cardiomyopathy

DCM is defined as a spectrum of heterogeneous myocardial disorders that are characterized by dilation of heart muscle, and the systolic impairment of the left or both ventricles in the absence of coronary artery disease pressure or volume overload [1]. The prevalence of DCM in the general population has been reported to range from 1/500 to 1/2500, although these numbers are widely questioned and may be underestimated [2]. Determining the true incidence and prevalence of DCM has been challenging, because of geographic variation, patient selection, and changes in diagnostic criteria. Variation likely reflects geographic and ethnic differences as well as the methodology used [3,4,5]. The global incidence is 7/100,000, with males being more frequently affected than females (3:1). In the pediatric population, DCM is the predominant type of cardiomyopathy, with an incidence of 0.57/100,000 cases [6,7]. DCM has high rates of morbidity and mortality; it is the third most frequent cause of heart failure and the most frequent type of cardiomyopathy [8,9]. Although DCM is the primary cause of heart transplant, the latter is usually a last resort for treating the disease given the limited availability of donor organs and complicated clinical course management [10]. The current diagnostic criteria for DCM are defined by the presence of (1) fractional shortening <25% (>2 SD) or ejection fraction <45% (>2 SD) and (2) left ventricle end-diastolic diameter >117% (>2 SD of predicted value of 112%, corrected for age, body surface area, and sex), excluding any known cause of myocardial disease [11]. The natural history of DCM has improved substantially over the past 10 years, with an incidence of major cardiac events below 2%, transplant free survival at eight years over 85%, and incidence of SCD below 0.5%, thanks to optimal medical therapy and cardiac devices [12]. However, there is a subgroup of patients with a high remaining risk of ventricular arrhythmias and SCD.

The underlying etiology of DCM seems to be crucial in improving management and long-term prognosis of these patients [13]. There are toxic, metabolic, inflammatory, and genetic mechanisms underlying non-ischemic DCM, which can exclusively affect the heart or concomitantly involve other organs under the same physiopathological process. Based on this, Pinto et al., classified idiopathic DCM into genetic and other non-genetic causes [11]. However, in many of the causes that are classified as non-genetic, such as in toxic enolic cardiomyopathy or peripartum cardiomyopathy, there is a shared genetic predisposition with both familial and sporadic idiopathic dilated cardiomyopathy and some of the genes that are implicated in DCM, as *TTN* truncated variants (*TTN*tv) and related genes, may also occasion these cardiomyopathies with specific triggers [14,15].

Multiple imaging modalities play a fundamental role in approaching a morphological and etiological diagnosis of DCM. Currently, echocardiography is the first-line imaging test to assess patients with DCM. It provides pivotal information not only for diagnosis, risk stratification, and treatment guidance, but also for screening family members [16]. It is a useful and accessible tool, albeit limited for characterizing preclinical stages of the disease as well as to identify the structural or functional patterns to establish the etiology of DCM [17]. Cardiac Magnetic Resonance (CMR) is the gold-standard test for the cardiomyopathies diagnosis, allowing for an integral evaluation of the heart’s bi-ventricular geometry, volumes, mass and function, tissue characterization (myocardial fat and edema), and the identification of focal or diffuse fibrosis and its quantification. It helps to distinguish primary from secondary forms of DCM and, specifically, to ascertain whether an ischemic etiology is present or identify other late gadolinium enhancement patterns.

However, nearly 50% of cases remain still without a conclusive etiology identified. In this group of idiopathic DCM cases, nearly 60% show a familial affectation, which suggests a rare genetic alteration as the cause of the disease. With the advent of high-quality next-generation sequencing (NGS) extended panels, the genetic causative variants can be identified in approximately 20% to 50% of all DCM cases, with a higher probability of finding a pathogenic alteration in the context of familial DCM -FDCM- (OR 1.52 (1.04-2.23), *p* = 0.03) [18].

FDCM is defined by the presence of (1) ≥2 affected relatives with DCM meeting the above criteria, or (2) when a first-degree relative of a diagnosed DCM patient dies inexplicably and suddenly before the age of 35 [19,20]. Currently, more than 60 genes have been associated with FDCM, mainly following an autosomal dominant inheritance [21]. Unlike hypertrophic cardiomyopathy, more restricted genetically, DCM is more heterogeneous [22]. The DCM-associated genes can be classified into functional groups: muscle contraction and cell structure and signaling, Ca^2+^ handling, and nuclear function [21]. *TTN* is the main gene that is currently associated with DCM, being responsible for nearly 40% of diagnosed DCM cases [23]. The *TTN* gene encodes the largest known human protein, called titin, a multi-functional sarcomeric structural protein that is specific to striated muscles [24]. Titin plays a major role in passive tension of cardiomyocytes and pre-mRNA undergoes extensive alternative splicing, leading to tissue-specific and developmentally regulated titin isoforms. Other genes that are associated to DCM are *LMNA, SCN5A*, *BAG3*, and *RBM20* [25]. Concretely, the *RBM20* gene is a crucial RNA-binding protein that controls the splicing of the *TTN* gene [26].

The increasing use of CMR in clinical practice, which depicts the appearance of the myocardium with excellent quality, has contributed to the detection of the left ventricle non-compaction (LVNC). A current debate is whether it represents a distinct pathology, a secondary phenotype that is associated to certain cardiomyopathies [27,28] or whether it may be a trait present in the general population with no specific prognostic value [29]. In a recent study conducted by Mazzaroto et al., the genetic architecture of left ventricular non-compaction revealed both substantial overlap with other cardiomyopathies, which indicated that, in many cases, LVNC belongs to a spectrum of more established cardiomyopathies, with non-compaction representing a phenotypic variation in these patients; and also a distinct etiology in a subset of cases. In this sense, truncating variants in *TTN* and *RBM20*, as well as non-truncating variants in the *RBM20* gene (within the pathogenic DCM hotspot of *RBM20*), were significantly enriched in both LVNC and DCM. In contrast, five variant classes were uniquely enriched in LVNC cases, of which truncating variants in *MYH7, ACTN2, PRDM16, RYR2,* and *HCN4* may represent a distinct LVNC etiology [30].

*RBM20* alterations have been observed in 2–3% of FDCM cases, and the altered expression of *RBM20* can shift the expression pattern of titin transcript variants, leading to cardiac diseases, such as FDCM [31]. Recently, alterations in *RBM20* have been associated with a severe arrhythmogenic phenotype in dilated cardiomyopathy (AR-DCM), leading to high risk for SCD [32].

In 2015, Spezzacatene et al., defined the AR-DCM in a cohort of 285 patients by the presence of ≥1 of the following: unexplained syncope, rapid non-sustained ventricular tachycardia (VT) (≥5 beats, ≥150 bpm), ≥1000 premature ventricular contractions/24 hour, and ≥50 ventricular couplets/24 hours, in the absence of overt heart failure. The AR-DCM subjects had a higher incidence of SCD, sustained VT and ventricular fibrillation (VF) when compared with non-AR-DCM patients (30.3% vs. 17.6%, *p* = 0.022), with no difference in death from congestive heart failure or heart transplantation [33]. Recently, Gigli et al., demonstrated that carriers of desmosomal and *LMNA* pathogenic variants experienced the highest rate of SCD/VT/VF, which was independent of the LVEF in a cohort of 487 DCM patients [34]. In a previous cohort with 269 carriers of pathogenic variants in the *LMNA* gene, the presence of LVEF <45%, non-sustained VT, non-missense *LMNA* mutations, and male condition were independent predictors SCD, resuscitation, and appropriate ICD treatment [35].

The current guidelines state that the prevention of SCD is based on the implantation of an implantable cardioverter-defibrillator (ICD), and it is recommended in all DCM patients with an ejection fraction (LVEF) below 35% and symptomatic heart failure [36]. ICDs have a high rate of complications (infection, mechanical complications as pneumothorax or bleeding, lead dysfunction, or inappropriate discharges) and, under the stated recommendation, they have not demonstrated to improve overall survival in primary prevention in a cohort of non-ischemic DCM patients from the DANISH study, where 74% of the patients had idiopathic DCM [37]. This trial confirmed that risk stratification in DCM is inadequate and needs revision. In addition, a subset of patients with DCM have a highly arrhythmogenic profile that exceeds the degree of morphological abnormalities and systolic dysfunction, as these patients may have arrhythmic manifestations prior to heart failure symptoms and they have a higher risk of SCD [38]. Genetic information and tissue characterization through CMR-LGE patterns may help to better characterize these patients. In this regard, gene-specific phenotypes of DCM have been recognized, and it is contemplated in clinical guidelines that an ICD should be considered in patients with DCM and a confirmed disease-causing *LMNA* mutation and the abovementioned clinical risk factors [35,36,39].

To date, few genes have been associated with AR-DCM, in addition to *LMNA*, also *SCN5A*, desmosomal pathogenic alterations and the specific phospholamban R14del pathogenic rare variant have been identified to confere greater arrhythmic risk [34,38,40]. Recently, two more genes, *FLNC* [41] and *RBM20* [32], have also been associated with AR-DCM, despite the lack of comprehensive analysis so far. For this subset of patients the decision for ICD implantation should be individualized and cautiously taken due to the lack of established recommendation. In this review, we focus on *RBM20*, a novel gene associated with aggressive arrhythmogenic phenotypes among DCM diagnosed patients. 

## 3. *RBM20*

**Figure 1:**
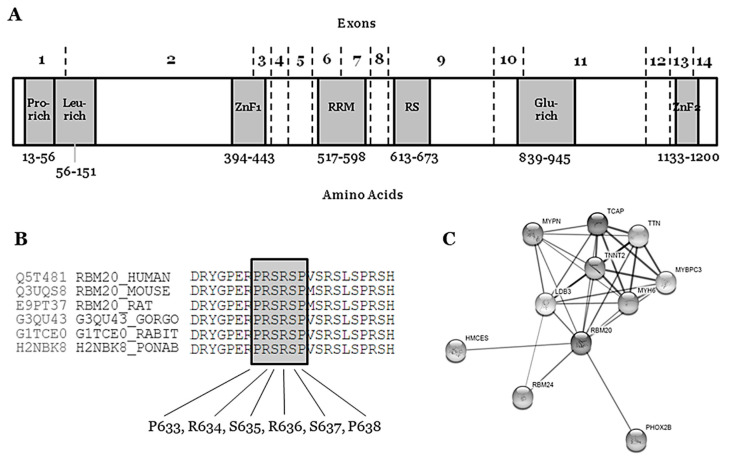
Structure and network of the RBM20 protein. (**A**) Glu-rich: Glutamate rich region; Leu-rich: Leucine rich region; Pro-rich: Proline rich region; RRM: RNA Recognition Motif; RS: Arginine-Serine Domain; ZnF1: Zinc Finger region 1; ZnF2: Zinc Finger region 2. (**B**) Conservation between species of RS region (amino acids 634–638). (**C**) Network of ten closest proteins to RBM20.

The *RBM20* gene (Gene ID: 282996; HGNC: 27424; OMIM: 613171; Gencode Gene: ENSG00000203867.7) is located on the long arm of chromosome 10 at position 25.2 (10q25.2) and it encodes the RNA binding motif protein-20 (RBM20). This gene comprises 14 exons (UniProtKB: Q5T481; RefSeq: NM_001134363; Gencode Transcript: ENST00000369519.3) that encode three conserved functional domains: two zinc finger (ZnF) domains and one RNA recognition motif (RRM)-type RNA-binding domain. In addition, sequence alignment from various vertebrate species shows three other conserved regions: a leucine (L)-rich region at the N-terminus, an arginine/serine (RS)-rich region just downstream from the RRM domain, and a glutamate (E)-rich region between the RS-rich region and ZnF2 domain [42] (Figure 1). The phosphorylation of arginine–serine–arginine–serine–proline residues in the RS region (RSRSP stretch) is necessary for RBM20 nuclear localization [43].

The *RBM20* gene is highly expressed during human fetal development (mainly 11–20 weeks of gestation) and in heart and skeletal muscle [44]. The protein (length: 1227 amino acids; mass: 134,357 Da) binds RNA and regulates the splicing of a subset of genes that are involved in cardiac development [45]. It is one of the few heart-specific splicing factors that regulate alternative splicing events of many genes, including *TTN* and *LDB3* [46,47], and it is associated with sarcomere assembly, ion transport, and diastolic function [26], as well as the expression of calcium handling, rendering high arrhythmic risk to *RBM20* carrier patients [48] (Figure 1).

In 2012, Gu et al., reported an animal model with a deficient titin splicing identifying a loss-of-function mutation in *RBM20* as the underlying cause for the pathological titin isoform expression. The affected rats had a 95 kb deletion that removed exons 2–14. The missing exons encode the RNA binding motif-, the RS-, and the Zn^2+^ finger domains. They determined that *RBM20* is required for titin splicing, as well as for the alternative splicing of many other conserved cardiac genes, as revealed by deep sequencing of rat and human samples. Defective splicing that is caused by the *RBM20* mutation in rats resulted in features resembling those of humans carrying *RBM20* mutations, including left ventricular dilatation, subendocardial fibrosis, arrhythmia, and sudden death. Adenoviral gene delivery to re-express *RBM20* in deficient cardiomyocytes was performed and reconstituted expression of the short titin isoform. They investigated an individual carrying a heterozygote p.Ser635Ala mutation in *RBM20* to validate *RBM20* dependent titin splicing and its relevance for human disease. On the protein level, the heterozygote *RBM20* mutation p.Ser635Ala shifted human titin isoform expression with an increased molecular weight that was similar to the larger isoform expressed in heterozygous rats [26].

## 4. Rare *RBM20* Variants in Familial Dilated Cardiomyopathy

Nowadays, individuals carrying *RBM20* pathogenic variants are at a high risk of AR-DCM and early ICD implantation should be discussed [49]. We performed an exhaustive review of the literature concerning *RBM20* and DCM published before October 2020. The data were collected from: HGMD (www.hgmd.org), ClinVar (www.ncbi.nlm.nih.gov/clinvar/intro), National Center for Biotechnology Information SNP database (www.ncbi.nlm.nih.gov/SNP), Index Copernicus (www.en.indexcopernicus.com), Google Scholar (scholar.google.es), Springer Link (www.link.springer.com), Science Direct (www.sciencedirect.com), Excerpta Medica Database (www.elsevier.com/solutions/embase-biomedical-research), and IEEE Xplore Digital Library (www.ieeexplore.ieee.org/Xplore/home.jsp). Genetic variants that were identified in articles were contrasted with variant data from Exome Variant Server (EVS, www.evs.gs.washington.edu/EVS) and Genome Aggregation Database (gnomAD, www.gnomad.broadinstitute.org), including recently added data regarding copy number variation (CNV). In addition, we consulted data for amino acid sequence and conservation between species in UniProt (www.uniprot.org). The variants were classified according to the American College of Medical Genetics and Genomics/Association for Molecular Pathology (ACMG/AMP) standards and guidelines for the interpretation of sequence variants [50] and described using the HGVS recommendations for the description of sequence variants [51,52]. Concerning frequency of disease-causing variants that are associated with rare inherited diseases, the vast majority of deleterious variants are extremely rare (<0.01%) [53]. ClinGen (www.clinicalgenome.org/), CardioClassifier (www.cardioclassifier.org), CardioBoost (www.cardiodb.org/cardioboost/), and VarSome (www.varsome.com) were consulted. Finally, all of the investigators discussed data and came to a consensus on the final classification of variants to avoid any bias.

In the *RBM20* gene, thirty-six rare non-synonymous variants have been reported as causes of FDCM. Of these, thirty-four are mapped within exons and two in intronic zones (c.1880 + 4_1880 + 6dupAGG and c.1528-1G>C -CI1516347 and CS183215 respectively-). Most of the exonic variants (32/34) are missense variants, and two are nonsense variants [p.(Arg688*) and p.(Gly1031*)—CM1516720 and CM1111136, respectively]. Importantly, all of the rare variants are reported in heterozygosis status. After comprehensive analysis of all clinical and genetic data published so far, five rare variants should be considered, likely benign (LB), eighteen of ambiguous role or variants of uncertain significance (VUS), nine as likely pathogenic (LP), and four as pathogenic (P) for FDCM (Table 1).

Rare variants that are definitely classified as LB can be discarded as causal for FDCM, mainly due to high frequency in the population. However, we cannot discard their potential role as phenotype modifiers. No VUS can be discarded as a potential cause of FDCM—a variant currently classified as VUS means that conclusive data do not exist, so additional studies are needed in order to clarify the definite role in FDCM. These 18 rare non-synonymous VUS in *RBM20* should be interpreted with caution by a group of experts, as clinical translation should be personalized, accounting for not only all published data, but also family segregation and phenotype of each patient [54]. The four rare variants classified as P are located in intron 5–6—c.1528-1G>C/IVS5asG>C-1—the end of exon 9—p.(Pro633Leu), p.(Arg688*)—and exon 11—p.(Gly1031*). These variants are considered definitely P due to their extremely low frequency in global population, in silico predictions and functional studies. Most of the variants classified as LP are located in exon 9 (RS domain, amino acids 634–638), suggesting a hot-spot for malignant arrhythmias in FDCM. Actually, a recent study identified two *RBM20* regions (exons 9 and 11) with a significant risk for cardiomyopathy, ventricular and atrial arrhythmias, and even SCD [32]. We only identified one rare variant classified as LP and located out of this hot-spot (p.Glu913Lys, Glutamic acid-rich domain). None of these LP variants can be classified as definitely P for FDCM mainly due to a lack of functional data. However, their highly malignant role is supported by low frequencies in global population databases as well as a conserved domain between species.

The hot-spot P-R-S-R-S-P between p.(Pro633) and p.(Pro638) contains crucial amino acids for the protein structure and function (Figure 1) (Table 1). In consequence, any amino acid modification inside this zone implies, a priori, a high probability of damaging effect in the protein structure and function. In the first amino acid of this hot-spot, only one rare variant has been recently reported­—p.(Pro633Leu). Clinical, genetic, and functional studies confirmed the pathogenic role of this rare variant [55]. Importantly, in this recent study, the authors also suggested that the upregulation of *RBM20* may be a viable therapeutic strategy for *RBM20*-related DCM. In the amino acid 634, two changes have been reported as LP in FDCM [p.(Arg634Trp) and p.(Arg634Gln), CM107456 and CM095004, respectively] [43,56]. In p.(Ser635), only one variation is reported—p.(Ser635Ala), CM125867 [26]. In p.(Arg636), three changes have been published as LP in FDCM—p.(Arg636Ser), p.(Arg636Cys), and p.(Arg636His) [56,57,58,59]. In the last two amino acids, p.(Ser637) and p.(Pro638), a change has been identified in each—p.(Ser637Gly) and p.(Pro638Leu) [60,61,62,63] (Figure 1) (Table 1). The establishment of hiPSC-CMs shows that pathogenic alterations in some of these amino acids may disorganize the sarcomeric complex [48,64,65]. All these variants were identified in families including aggressive arrhythmogenic phenotypes, occasionally in individuals with discrete structural heart alterations, and with a high penetrance of the disease. Despite this fact, we cannot discard that future studies and additional evidence may allow for the identification of other rare variants that are located in different regions of *RBM20* and still not associated with arrhythmogenic phenotypes in FDCM. Finally, with regard to CNVs as being potentially responsible for FDCM, to date no structural alteration has been identified in *RBM20*. Only two CNVs have been associated with FDCM, one located in *LMNA* [66] and the other in *BAG3* [67], explaining <5% of FDCM cases together [68,69].

The first pathogenic variant in the *RBM20* gene was reported in 2009 as a novel cause for familial DCM [56]. In this first cross-sectional study, pathogenic variants in *RBM20* were present in the DCM families showing high penetrance, a tent to young age at diagnosis, a notable presence of end-stage heart failure, and high mortality, according to the available information in the included individuals [57]. Nowadays, nearly 30 rare variants in *RBM20* have been reported, explaining 2–3% of DCM [18,70] and supporting an aggressive arrhythmogenic phenotype with a higher risk of SCD [32,58,61,71,72] (Table 2).

Reefat et al., studied 283 individuals with DCM from the GRADE cohort (that studies the genetic background of patients carrying an ICD), including only non-transplanted patients and with no heart assist device [61]. The patients were screened for *RBM20* pathogenic alterations. The mean age of subjects with DCM was 58 ± 13 years, 64% were males, and the mean follow-up time was 24.2 ± 17.1 months after ICD placement. Pathogenic alterations in *RBM20* were identified in eight subjects with DCM (2.8%). Carriers of these pathogenic rare variants had a similar survival, transplantation rate, and frequency of ICD therapy as compared with non-variant carriers. Three of eight subjects carrying *RBM20* alterations (37.5%) had atrial fibrillation (AF), whereas 19 subjects without rare pathogenic alterations (7.4%) had AF (*p* = 0.02) (Table 2). Among all of the GRADE subjects, rs35141404, a common genetic polymorphism situated in the *RBM20* gene, was associated with AF (*p* = 0.006), and this association remained in the subset of GRADE subjects with DCM (*p* = 0.047) [61]. This locus have been validated by large GWAS AF studies in general population [73,74]. Haas et al., investigated gene groups in DCM patients to identify genotype-phenotype correlations. In a cohort of 639 patients, the presence of P, LP or VUS *RBM20* variants identified in 15 patients provided an OR of 5.65 (1.89–16.86; *p* = 0.002) for ICD carrier status in DCM [18]. Van den Hoogenhof et al., compared 18 DCM patients carrying *RBM20* alterations to 22 DCM patients carrying *TTN* alterations, and found that 44% of patients carrying any *RBM20* alteration had sustained ventricular arrhythmias (Sustained VT or VF) when compared to 5% of patients carrying any *TTN* alteration, despite similar LVEF. No differences in non-sustained VT and AF prevalence were detected [72] (Table 2). In another multicenter registry, 72 DCM patients carrying alterations in the *RBM20* gene were compared to idiopathic DCM (*n* = 633), *TTN*tv related to DCM, and *LMNA*-related DCM. There was a considerable family history of SCD (51%) similar to *LMNA* patients (44%, *NS*) and greater than idiopathic DCM and *TTN*tv DCM patients (15% in both, *p* < 0.001 respectevily). The carriers of *RBM20* alterations were more likely to have sustained VT (25%) than idiopathic DCM cohort (2%, *p* < 0.001) and *TTN*tv variants cohort (1%, *p* < 0.001) and, similar to *LMNA* patients (21%, *NS*), defined as sustained VT or VF on monitoring for idiopathic DCM, TTNtv, and *LMNA,* and as sudden cardiac arrest or ICD discharge for *RBM20* [32] (Table 2). In the largest cohort of *RBM20* patients published to date, Hey et al., included a total of 80 individuals from 15 families carrying pathogenic alterations (p.Arg634Gln, p.Arg636His, p.Arg636Ser, p.Pro638Leu, and p.Glu913Lys) (Table 1 and Table 2). Ten index-patients were shown to carry the same p.Arg636Ser and seven of those shared the haplotype analyses, which suggested a common founder. The penetrance was 66% (53/80) and age-dependent. The males were both significantly younger and had lower ejection fraction at diagnosis than females (age, 29 ± 11 versus 48 ± 12 years; *p* < 0.01; ejection fraction, 29 ± 13% versus 38 ± 9%; *p* < 0.01). Subsequently, 11 of 31 affected males needed a cardiac transplant while none of 22 the affected females required this treatment (*p* < 0.001). However, two females died of end-stage heart failure at the age of 54 and 72, while none of the males did. Remarkably, seven young males and no females developed DCM in their teens, requiring heart transplant in four individuals before the age of 19 (p.Arg636His, p.Glu913Lys, p.639Leu). Thirty percent of *RBM20*-carriers with DCM (16/53) died suddenly or experienced severe ventricular arrhythmia: six died suddenly, three were successfully resuscitated from a cardiac arrest because of VF, two had episodes of sustained VT requiring cardioversion, and five received appropriated therapies by a prophylactic ICD. From the six patients that died suddenly, 1 SCD occurred in a patient with a LVEF of 30% and 5/6 SCD patients were diagnosed of DCM by post-mortem autopsy, belonging four of these cases to one of the two families carrying the same *RBM20* p.Arg636Ser variant. The only available pathology report described a dilated LV and a weight of 475 g (0.8% of her body weight). For the eleven patients experiencing ventricular arrhythmia with available echocardiography, the median LVEF was median 30%; range, 10–47%. However, 36% (4/11) had a LVEF >30%. No adverse events were identified among healthy *RBM20*-carriers with a normal cardiac investigation. The event-free survival of male *RBM20*-carriers was significantly shorter when compared with female carriers (*p* < 0.001). [71] (Table 2).

Taking all of the data into account, *RBM20* pathogenic alterations may cause disturbing cardiac contraction and impair cardiac conduction [57]. The mean age at diagnosis is around the forth decade. LVEF is usually impaired, leading to mild to severe DCM, which can lead to heart failure and eventually heart transplantation, which can be required at very young ages. In the published series, no fatal events have been detected in patients without LV structural abnormalities. However, some of these patients have been diagnosed post-mortem, as SCD was the first manifestation of the disease. Similarly to *LMNA* rare variants, the disease expression seems to be gender-specific. Most of *RBM20* carrier’s males are diagnosed at younger ages and suffer major cardiac events before 40 years old when comparing to females (less than 5%) [71]. Lower LVEF justifies the increased level of SCD and heart transplant in males. Analysis of human induced-pluripotent stem cell (hiPSC)-derived cardiomyocytes (hiPSC-CMs) from DCM patients carrying rare variants in *RBM20* have shown that pathogenic alterations in this gene may disorganize the sarcomeric complex [48,64]. The RBM20 hiPSC-CMs were defective in calcium handling machinery with prolonged levels in the cytoplasm and higher spike amplitude [48,64]. Indeed, this fact supports the malignant arrhythmogenic nature of the rare alterations in this gene [50], thereby requiring patients carrying these variants to be more closely followed together with the adoption of personalized preventive measures.

The stratification of SCD risk has been precluded by the use of heterogeneous subsets of patients with idiopathic DCM and by the use of risk models in which predictions are based on static parameters disregarding the disease course. Furthermore, it is important to consider that a large proportion of the *RBM20*-DCM patients published in the literature come from the same families, representing phenotypes of specific rare pathogenic alterations and possibly with an additional common genetic background that may add information to the aggressiveness of the disease. Similarly to other cardiomyopathies, disease expression may vary depending on site-specific alterations in *RBM20*, so accounting that information in future projects will bring clinical value to such work. Of note is that the aggressiveness of the disease has been demonstrated differently according to biological sex, which suggests that a different clinical approach should be applied accordingly. The current approach to personalized risk stratification for DCM is shifting towards a better characterization of the underlying etiology of DCM, in which genetic study has a paramount value. Family screening is mandatory among patients in order to identify asymptomatic DCM affected individuals at risk of SCD, which can be the first symptom of the disease.

## 5. Conclusions

Rare non-synonymous deleterious alterations in *RBM20* are associated with aggressive arrhythmogenic phenotypes, and early ICD implantation should be recommended. Although these harmful alterations may be distributed throughout the gene, most highly deleterious variants that have been reported so far are located in the RS domain, suggesting a hot-spot that is highly associated with malignant events. Early identification of potentially pathogenic rare variants in *RBM20* may help to promote the adoption of preventive personalized measures to reduce the risk of lethal arrhythmias among affected individuals.

## Figures and Tables

**Table 1:** Data of variants in *RBM20* potentially associated with Dilated Cardiomyopathy.

Nucleotide Change	Protein Change	dbSNP	gnomAD (MAF%)	ClinVar (Disease)	HGMD (Disease)	CC	ACMG Score	RBM20 Domain	Arrhythmogenic Phenotype
c.247C > A	p.(Leu83Ile)	rs536357058	1/155140 (0.0006%)	VUS (DCM)	CM1111132	VUS	VUS	Exon 2	Yes
					(DM; DCM)			Leucine-rich region	
c.680G > T	p.(Gly227Val)	rs202238753	225/185204 (0.12%)	LB (DCM)	CM1821953	LB	VUS	Exon 2	No
					(DM; DCM)				
c.769A > G	p.(Thr257Ala)	rs1418674149	1/153900 (0.0006%)	NA	CM1815813	VUS	VUS	Exon 2	Yes
					(DM; DCM)				
c.1175G > A	p.(Arg392Gln)	rs751788298	3/185862 (0.0016%)	NA	CM1815814	VUS	VUS	Exon 2	NA
					(DM; DCM)				
c.1364C > T	p.(Ser455Leu)	rs189569984	862/153884 (0.56%)	LB	NA	LB	LB	Exon 4	No
c.1494C > A	p.(Ser498Arg)	rs774916799	2/153882 (0.0013%)	VUS (DCM)	CM1815816	VUS	VUS	Exon 4	Yes
					(DM; DCM)				
c.1528-1G > C	-	rs534513476	NA	NA	CS183215	VUS	P	Intron 5–6	Yes
					(DM; DCM)				
c.1603G > A	p.(Val535Ile)	rs183007628	6/188686 (0.0031%)	VUS (DCM)	CM107458	VUS	VUS	Exon 6	Yes
					(DM; DCM)			RNA Recognition Motif	
c.1760T > A	p.(Leu587His)	NA	NA	NA	CM1815817	VUS	VUS	Exon 7	Yes
					(DM; DCM)			RNA Recognition Motif	
c.1764T > G	p.(Ile588Met)	NA	NA	NA	CM183216	VUS	VUS	Exon 7	Yes
					(DM; DCM)			RNA Recognition Motif	
c.1880 + 4_1880 + 6dupAGG	-	rs1227694990	200/187706 (0.1%)	LB (DCM)	CI1516347	VUS	VUS	Intron 7−8	No
					(DM; DCM)				
c.1898C > T	p.(Pro633Leu)	rs747880281	1/151498 (0.0006%)	VUS (DCM)	NA	VUS	P	Exon 9	Yes
								Arginine-Serine Domain	
c.1900C > T	p.(Arg634Trp)	NA	NA	NA	CM107456	VUS	LP	Exon 9	Yes
					(DM; DCM)			Arginine-Serine Domain	
c.1901G > A	p.(Arg634Gln)	rs267607001	1/152378 (0.0006%)	P (DCM)	CM095004	VUS	LP	Exon 9	Yes
c.1901G > T	p.(Arg634Leu)				(DM; DCM)			Arginine-Serine Domain	
c.1903T > G	p.(Ser635Ala)	NA	NA	NA	CM125867	VUS	LP	Exon 9	Yes
					(DM; DCM)			Arginine-Serine Domain	
c.1906C > A	p.(Arg636Ser)	rs267607002	NA	NA	CM095005	LP	LP	Exon 9	Yes
					(DM; DCM)			Arginine-Serine Domain	
c.1906C > T	p.(Arg636Cys)	rs267607002	NA	NA	CM107457	LP	LP	Exon 9	Yes
					(DM; DCM)			Arginine-Serine Domain	
c.1907G > A	p.(Arg636His)	rs267607004	NA	NA	CM095006	VUS	LP	Exon 9	Yes
					(DM; DCM)			Arginine-Serine Domain	
c.1909A > G	p.(Ser637Gly)	rs267607005	NA	NA	CM095007	VUS	LP	Exon 9	Yes
					(DM; DCM)			Arginine-Serine Domain	
c.1913C > T	p.(Pro638Leu)	rs267607003	NA	NA	CM095008	VUS	LP	Exon 9	Yes
					(DM; DCM)			Arginine-Serine Domain	
c.1997G > A	p.(Arg666Gln)	rs202011408	5/154830 (0.003%)	NA	CM1716804	VUS	VUS	Exon 9	Yes
					(DM; DCM)			Arginine-Serine Domain	
c.2021A > G	p.(Asp674Gly)	rs1475557145	1/155286 (0.0006%)	VUS (DCM)	NA	VUS	VUS	Exon 9	NA
c.2042A > G	p.(Tyr681Cys)	rs372048968	23/186630 (0.01%)	VUS (DCM)	CM1815818	LB	LB	Exon 9	No
					(DM; DCM)				
c.2062C > T	p.(Arg688Ter)	rs794729150	1/31344 (0.003%)	VUS (DCM)	CM1516720	VUS	P	Exon 9	Yes
					(DM; DCM)				
c.2109G > C	p.(Arg703Ser)	rs988797559	2/186026 (0.001%)	NA	CM1111134	VUS	VUS	Exon 9	Yes
					(DM; DCM)				
c.2147G > A	p.(Arg716Gln)	rs375798246	21/155108 (0.013%)	VUS (DCM)	NA	LB	LB	Exon 9	No
c.2282G > A	p.(Arg761Gln)	rs556897484	4/156496 (0.002%)	NA	NA	VUS	VUS	Exon 9	NA
c.2662G > A	p.(Asp888Asn)	rs201370621	603/155726 (0.3%)	VUS (DCM)	NA	LB	LB	Exon 11	No
c.2737G > A	p.(Glu913Lys)	rs397516607	NA	LP (DCM)	NA	LP	LP	Exon 11	Yes
c.2741T > C	p.(Val914Ala)	rs794729154	NA	NA	NA	VUS	VUS	Exon 11	Yes
c.2714T > A	p.(Met950Lys)	NA	NA	NA	NA	VUS	VUS	Exon 11	NA
c.3091G > T	p.(Gly1031Ter)	rs794729157	NA	NA	CM1111136	VUS	P	Exon 11	Yes
					(DM; DCM)				
c.3115C > T	p.(Pro1039Ser)	rs727503392	40/188260 (0.02%)	LB (DCM)	CM1815819	VUS	LB	Exon 11	No
					(DM; DCM)				
c.3242C > G	p.(Pro1081Arg)	rs1268330519	NA	NA	CM1111137	VUS	VUS	Exon 12	Yes
					(DM; DCM)				
c.3545G > A	p.(Arg1182His)	rs563762318	47/185298 (0.025%)	LB (DCM)	CM1510988	VUS	VUS	Exon 12	Yes
					(DM; DCM)			Zinc Finger domain 2	
c.3616G > A	p.(Glu1206Lys)	rs757389650	8/181254 (0.004%)	VUS (DCM)	CM1111138	VUS	VUS	Exon 14	NA
					(DM; DCM)				

ACMG: American College of Medical Genetics and Genomics, CC: Cardio Classifier, ClinVar: Clinical Variation, DCM: Dilated Cardiomyopathy, DM: Disease Mutation, gnomAD: Genome Aggregation Database, HGMD: Human Genome Mutation Database, LB: Likely Benign, LP: Likely Pathogenic, MAF: Minor Allele Frequency, NA: No data Available, P: Pathogenic, and VUS: Variant of Uncertain Significance.

**Table 2:** Rare variants in *RBM20*.

	Brauch et al., 2009 (*n* = 39, DCM) ^NC^	Li et al., 2010 (*n* = 16, DCM) ^NC^	Refaat et al., 2012 (*n* = 8, DCM)	Wells et al., 2013 (*n* = 19 carriers) ^NC^	Van den Hoogenhof et al., 2018 (*n* = 18, DCM)	Hey et al., 2019 (*n* = 53, DCM)	Parikh et al., 2019 (*n* = 74, carriers)
Age diagnosis	36 ± 13.2	37.6 ± 9	-	33.8 ± 11.5	42 ± 14	37 ± 15 ^&^	37 ± 15 ^†^
Males	19 (49%)	8 (50%)	4 (50%)	14 (82%)	8 (44%)	31 (58%)	-
Follow-up (months)	60 (12−204)	-	27.4 ± 15.7	-	71 ± 65	86 (24−150)	-
Mean LVEF	35.3 ± 11.5	29.3 ± 8.6	-	48.8 ± 13	37 ± 17	32 ± 12 ^&&^	40 ± 17
FH SCD	39 (100%)	-	-	-	13 (72%)	-	22/43 (51%) ^††^
NSVT	-	1 (6%)	-	-	5 (28%)	-	21/59 (36%)
Sustained VT or VF	9 (23%)	1 (6%)	0	-	8 (44%) ^¶^	11 (21%) ^&&&^	-
ICD therapy	-	0	1 (12.5%)	-	-	-	9/32 (28%) ^†††^
SCD	3 (7.7%)	1 (6%)	0	-	-	6 (11.3%) ^&&&^	5/60 (8%)-SCA ^†††^
AF	3 (7.7%)	2 (12.5%)	3 (37.5%) *		6 (33%)	-	10/58 (17%) ^††††^
HTx	4 (mean age 28.5)	2 (12.5%)	1 (12.5%)	1 (5.2%, 17 years old) ^+^	-	11 (21%) ^&&&&^	5/74 (7%) ^NC^
Death	11 (28%, mean age 45): 4 HF (mean age 54.7), 3 SCD (mean age 39)	3 (11.5%)	0	11 (57.9%) ^+^	-	2 (4%, end-stage HF at 54 and 73 years old)	3/74 (4%) ^NC^

Clinical characteristics of patients with variants in the *RBM20* gene described in the series published to date. * Significantly different compared to the control group in the study, 275 all-cause DCM patients’ non *RBM20* variants carriers. ^+^ Mean age transplant or death 46.1 ± 17.3 years old. ^¶^ Significantly different as compared to the control group: twenty-two DCM patients with *TTNtv*. Hey et al., did comparisons separating for individual’s sex. Thirty-one DCM male patients and fifty-one DCM female patients carrying *RBM20* variants were compared to thirty DCM male patients and forty-nine DCM female patients of unknown cause. ^&^ Males were diagnosed at younger age compared to controls and females at older age compared to controls. ^&&^ Males had lower EF at diagnosis compared to control group. ^&&&^ Mean age of first ventricular arrhythmia (VA) including SCD was 44 ± 14 years. ^&&&&^ Females with *RBM20* variants were less transplanted than the control group. However, two of them died of end-stage heart failure at the age of 44 and 73 while none males did. Parikh et al., compared the forty-three index cases from their study to 663 all-cause DCM patients, eighty-three *TTNtv* variants DCM patients, and eighty-seven *LMNA* DCM patients. ^†^ *RBM20* patients were diagnosed at younger age and ^††^ had greater FH of SCD than all cause-DCM and *TTNtv*-DCM patients. ^†††^ *RBM20* patients had greater incidence of sustained VA defined as sustained VT or VF on monitoring for DCM *TTNtv* and *LMNA* and as SCA or ICD discharge for *RBM20*. ^††††^ *RBM20* prevalence of atrial fibrillation showed non-significant difference to all-cause a *TTNtv* DCM patients, and was lower compared to *LMNA* patients. ^NC^ Not compared to a control group, LVEF: Left-ventricle ejection fraction, FH SCD: Family history of sudden cardiac death, NSVT: non-sustained ventricular tachycardia, ICD: Implantable cardiac defibrillator, SCD: Sudden cardiac death, AF: Atrial fibrillation, HTx: Heart transplant, HF: Heart Failure, and SCA: Sudden cardiac arrest.

## Data Availability

All the data included in the present study is available in the cited original articles and the databases specified in the manuscript. Please see Methods section.

## Abbreviations

ACMG	American College of Medical Genetics and Genomics
AR-DCM	Arrhythmogenic phenotype in dilated cardiomyopathy
CC	Cardio Classifier, ClinVar: Clinical Variation
CMR	Cardiac Magnetic Resonance
CNV	Copy Number Variation
DM	Disease Mutation
DCM	Dilated Cardiomyopathy
ECG	Electrocardiogram
FDCM	Familial DCM
gnomAD	Genome Aggregation Database
HGMD	Human Genome Mutation Database
hiPSC-CMs	human induced-pluripotent stem cell-derived cardiomyocytes
ICD	Implantable Cardiac Defibrillator
LB	Likely Benign
LP	Likely Pathogenic
MAF	Minor Allele Frequency
NGS	Next Generation Sequencing
P	Pathogenic
SCD	Sudden Cardiac Death
SD	Standard Deviation
VF	Ventricular Fibrillation
VT	Ventricular Tachycardia
VUS	Variant of Uncertain Significance
